# Tomographic X-ray scattering based on invariant reconstruction: analysis of the 3D nanostructure of bovine bone

**DOI:** 10.1107/S1600576721000881

**Published:** 2021-03-03

**Authors:** Paolino De Falco, Richard Weinkamer, Wolfgang Wagermaier, Chenghao Li, Tim Snow, Nicholas J. Terrill, Himadri S. Gupta, Pawan Goyal, Martin Stoll, Peter Benner, Peter Fratzl

**Affiliations:** a Department of Biomaterials, Max Planck Institute of Colloids and Interfaces, Am Mühlenberg 1, 14476 Potsdam, Germany; b Diamond Light Source Ltd, Diamond House, Harwell Science and Innovation Campus, Didcot, Oxfordshire OX11 0DE, United Kingdom; c School of Engineering and Materials Science, Queen Mary University of London, London E1 4NS, United Kingdom; d Max Planck Institute for Dynamics of Complex Technical Systems, Sandtorstrasse 1, 39106 Magdeburg, Germany; e Department of Mathematics, TU Chemnitz, Reichenhainer Strasse 41, 09126 Chemnitz, Germany

**Keywords:** small-angle X-ray scattering, SAXS, tomography, bovine bone, fibrolamellar unit, *T* parameter, scattering tomography, fibrolamellar bone

## Abstract

Small-angle X-ray scattering (SAXS) is an effective characterization technique for multi-phase nanocomposites. The structural complexity and heterogeneity of biological materials require the development of new techniques for the 3D characterization of their hierarchical structures. Emerging SAXS tomographic methods allow reconstruction of the 3D scattering pattern in each voxel but are costly in terms of synchrotron measurement time and computer time. To address this problem, an approach has been developed based on the reconstruction of SAXS invariants to allow for fast 3D characterization of nanostructured inhomogeneous materials. SAXS invariants are scalars replacing the 3D scattering patterns in each voxel, thus simplifying the 6D reconstruction problem to several 3D ones. Standard procedures for tomographic reconstruction can be directly adapted for this problem. The procedure is demonstrated by determining the distribution of the nanometric bone mineral particle thickness (*T* parameter) throughout a macroscopic 3D volume of bovine cortical bone. The *T* parameter maps display spatial patterns of particle thickness in fibrolamellar bone units. Spatial correlation between the mineral nano­structure and microscopic features reveals that the mineral particles are particularly thin in the vicinity of vascular channels.

## Abstract 2

A new tomographic approach using X-ray scattering is presented, allowing the characterization of the 3D nanostructure of hybrid materials.

## 1. Introduction

Many biological materials incorporate nanoscopic mineral particles into an organic matrix, thereby reconciling conflicting material properties like strength and toughness (Weinkamer & Fratzl, 2016 ▸; Ritchie, 2011 ▸; Meyers *et al.*, 2008 ▸). From both a biomedical (Xi *et al.*, 2018 ▸; Milovanovic *et al.*, 2015 ▸) and bio-inspired materials (Bouville *et al.*, 2014 ▸; Studart, 2013 ▸) perspective, an important example is bone, which is a nanocomposite of stiff inorganic apatite particles embedded in a softer collagenous matrix (Weiner & Wagner, 1998 ▸; Fratzl & Weinkamer, 2007 ▸). The mineral particles in bone have roughly the shape of thin and elongated platelets with a thickness of 2–5 nm.

The characteristics of the mineral particles not only influence the mechanical performance but also provide hints about changes in bone physiology (Pathi *et al.*, 2011 ▸). Previous work showed that the aspect ratio and staggered arrangement of the mineral particles affect the mechanical properties of bone (Jäger & Fratzl, 2000 ▸; Xi *et al.*, 2018 ▸; Bar-On & Wagner, 2013 ▸). In general, the thickness of the mineral particles can be viewed as an indication of tissue age and normally correlates with the degree of mineralization (Zizak *et al.*, 2003 ▸; Roschger *et al.*, 2001 ▸; Fratzl *et al.*, 1991 ▸), except in the case of *osteogenesis imperfecta*, the brittle bone disease (Fratzl-Zelman *et al.*, 2014 ▸). He *et al.* (2017 ▸) found that regions affected by cancer metastasis in mouse models contain thinner and less oriented mineral particles compared with healthy bone.

The high contrast in electron density between the inorganic and organic components in bone makes scattering techniques an attractive approach to characterize the mineral particles (Rinnerthaler *et al.*, 1999 ▸; Pabisch *et al.*, 2013 ▸). A particularly powerful approach is 2D scanning small-angle X-ray scattering (SAXS), where an X-ray beam is used to scan the sample, providing maps of the local mineral nanostructure with a spatial resolution of several micrometres or even less (Pabisch *et al.*, 2013 ▸; Paris *et al.*, 2000 ▸). The data obtained in this way are 4D, with two real-space dimensions corresponding to the mapping by scanning of the X-ray beam, and another two from the 2D SAXS patterns that correspond to planar sections through reciprocal space.

For a number of research questions a higher-dimensional mapping of mineral characteristics would be desirable. A way of increasing the dimensionality of the information is to collect 3D scattering patterns using a thin sample, but measuring the scattering signal under different angles by rotating the sample. Here the data are quasi-2D in real space but with three dimensions in reciprocal space. Combined SAXS and wide-angle X-ray scattering (WAXS) have been used to investigate the crystalline and morphological texture of mineral particles in human vertebrae, showing a close relationship between the *c*-axis orientation and the orientation distribution of the mineral platelets, the plate normal being perpendicular to the *c* axis (Jaschouz *et al.*, 2003 ▸). In that study, it was shown that the mineral platelets are aligned with the collagen fibers along the trabecula axis. In synchrotron scanning SAXS/WAXS with a beam size of 1 µm, it was demonstrated that mineral platelets in human osteonal bone change their orientation over a length scale of approximately the thickness of a lamella of ∼5–10 µm (Seidel *et al.*, 2012 ▸; Wagermaier *et al.*, 2006 ▸), in agreement with the previously proposed rotated twisted plywood structure (Weiner *et al.*, 1999 ▸). This technique can be combined with serial sectioning and scanning of slices under various angles. The result of one such experiment was a full 6D data set with a 3D map of 3D SAXS patterns for a human trabecula (Georgiadis *et al.*, 2016 ▸), whereby results about ultrastructural 3D orientation were confirmed using polarized light microscopy (Georgiadis *et al.*, 2015 ▸).

An alternative approach to serial sectioning is SAXS tomography. Instead of reconstructing the attenuation coefficients as in standard microcomputed tomography, the aim here is to measure a bulk sample under different directions of the beam and to reconstruct 3D SAXS patterns in a 3D volume. Under the assumption of structural isotropy, reconstructions of the 1D SAXS pattern have been performed for samples like injected polymers (Schroer *et al.*, 2006 ▸), nanoporous glass (Feldkamp *et al.*, 2009 ▸) and rat brains (Jensen *et al.*, 2011 ▸). More recently, the full reconstruction of the spatially heterogeneous anisotropic ultrastructure in bone (Liebi *et al.*, 2015 ▸) and tooth (Schaff *et al.*, 2015 ▸) has been achieved.

In SAXS measurements the sample is rotated around many different rotational axes. The reconstruction of the obtained 6D data set is computationally intensive and becomes tractable by assuming certain symmetries in the data (tensor tomography) and by fitting the 3D pattern by spherical harmonics (Liebi *et al.*, 2015 ▸). Another approach also preserves oriented scattering information by the introduction of virtual scattering axes (Schaff *et al.*, 2015 ▸). The application of regularization strategies during reconstruction can save experimental data acquisition time (Liebi *et al.*, 2018 ▸) and stronger assumptions on the symmetry of the SAXS pattern can substantially reduce the time needed for reconstruction (Gao *et al.*, 2019 ▸).

However, in many instances the full oriented 3D SAXS pattern is not required in every voxel of the reconstructed volume. Instead, information derived from the SAXS patterns such as a Porod-like or Guinier-like analysis (Jensen *et al.*, 2011 ▸) is sufficient. Here we develop an approach to reconstruct directly the spatial variation of the particle thickness parameter instead of the spatial variation of full 3D-SAXS patterns. While the original data to be reconstructed consist of a 3D reciprocal-space picture in each real-space voxel, using invariants of the small-angle scattering replaces the 3D-SAXS data by scalars. This effectively reduces the problem to the reconstruction of a 3D matrix of scalars as in conventional X-ray absorption tomography and allows the use of efficient algorithms that have previously been developed for micro­computed tomography.

**Figure 1:**
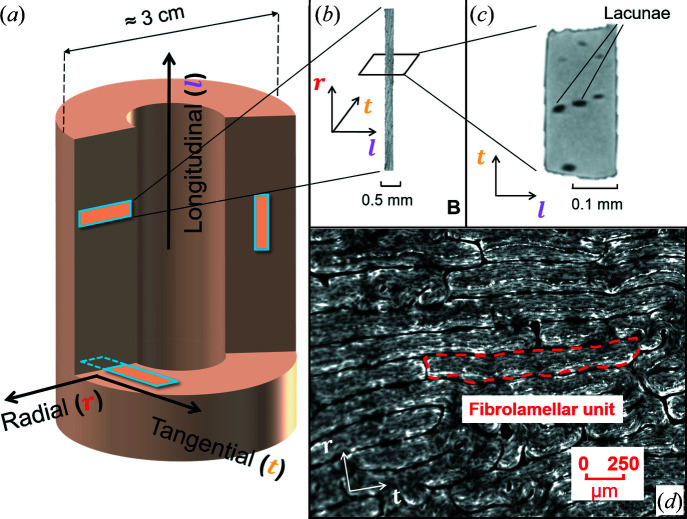
Macro- and microstructure of femoral bovine bone. (*a*) A representation of femoral bovine bone at the macroscale, showing the directions of the long axes (radial, tangential and longitudinal) of the measured samples. (*b*) A µCT reconstruction of a sample with the long axis aligned to the radial direction of the femur, with an enlargement of a section shown in (*c*). (*d*) A light microscopy image showing the arrangement of fibrolamellar units at the microscale. The red dashed line outlines one such unit.

To test our approach, we chose bovine fibrolamellar bone as our model system, mainly for structural reasons: (i) the cortex of a long bone is structurally anisotropic with a preferred direction along the long axis of the femur, (ii) the bone consists of microscopic fibrolamellar units and these units are arranged in a regular brick-like fashion, with the shortest dimension oriented towards the bone center with a thickness of approximately 200 µm (Fig. 1 ▸), and (iii) the fibro­lamellar unit contains structural features (different layers like the parallel-fibered layer and hypercalcified layer, or vascular channels) that are large enough to be resolved with our beam size of 10 µm. The structural anisotropy of the fibrolamellar bone is reflected in extremely anisotropic mechanical properties, with the elastic modulus being 20 times higher along the fiber direction than perpendicular to it (Seto *et al.*, 2008 ▸). In the transition zone to the neighboring fibrolamellar unit, lamellar bone is found, which again has a preferred fiber orientation along the bone long axis and contains blood vessels. Within both fibrolamellar and lamellar bone a porous network is located. The lacunae of this lacuno­canalicular network accommodate the cell bodies of osteocytes, while their cell processes run in canaliculi. In fibrolamellar bone of minipigs, the general orientation of the canaliculi was found to be radial with tortuous and twisted pathways (Magal *et al.*, 2014 ▸).

With the simplified reconstruction approach described in this paper, we were able to reconstruct spatial distributions of mineral particle characteristics in bovine fibrolamellar bone consisting of woven bone layers augmented by lamellar layers. A spatial correlation between the mineral nanostructure and microscopic features like vascular channels demonstrated that mineral particles are particularly thin in their vicinity.

## 2. Materials and methods

### 2.1. Samples

Four matchstick-like samples of bovine bone were used for both microcomputed tomography (µCT) and synchrotron scattering measurements (Fig. 1 ▸). All the samples were obtained from the femur of a 23 month old cow, obtained from a slaughterhouse. The mid-part of the diaphysis was cut into approximately 2 cm thick pieces, the endosteal cancellous bone was removed from the slice and the samples were stored at 251 K. Using a low-speed saw (Buehler Isomet, Düsseldorf, Germany) under water cooling, plate-like samples were cut under three different orientations, with the normal to the plate pointing in the radial, longitudinal and tangential directions, respectively. Each plate was then polished to roughly 150 µm thickness. The plates were cut again to obtain stick-like samples of approximately 4 mm in length [Fig. 1 ▸(*b*)] with a rectangular section of about 250 × 150 µm [Fig. 1 ▸(*c*)]. The long axes of the stick-like samples were aligned with one of the main directions of the femoral bovine bone [Fig. 1 ▸(*a*)], and the samples are, therefore, referred to as radial (two samples), longitudinal (one sample) and tangential (one sample).

### 2.2. Micro-computed tomography

µCT measurements of all four samples were performed with an EasyTom 160 (RX solutions, Chavanod, France). In each measurement, the applied tube voltage was 60 kV and the integration times (duration of each tomographic projection) 11.0 s, resulting in a voxel size of 1.39 µm^3^.

### 2.3. Synchrotron measurements

Two different synchrotron SAXS experiments were performed, on the µSpot beamline at BESSY II (Germany) (Paris *et al.*, 2007 ▸) and on the I22 beamline at Diamond Light Source (DLS, UK) (Smith *et al.*, 2019 ▸). Throughout this paper, reported values of the experimental settings are separated by a forward slash (/), where the first value refers to BESSY II and the second to DLS. The monochromatic X-ray beam had an energy of 18 keV/14 keV and a beam size of 20 µm/10 µm, defined by a pinhole/secondary source slits. The sample-to-detector distance was about 300 mm/5495 mm. Scattered signals were acquired by an EIGER X 9M/Pilatus P3-2M detector with an exposure time of 5 s/0.5 s.

**Figure 2:**
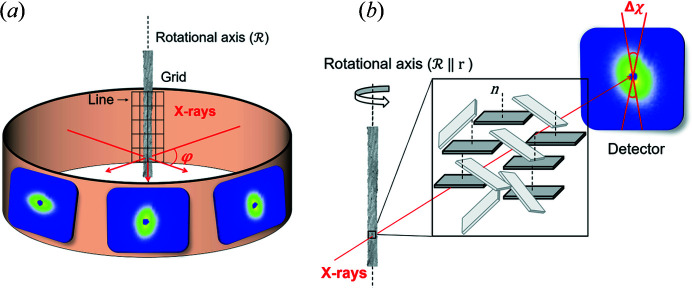
The experimental setup adopted for the synchrotron measurements. (*a*) A schematic diagram showing SAXS signals acquired at different rotational angles φ. (*b*) A µCT reconstruction of the sample with the rotational axis aligned to the radial direction shown in Fig. 1 ▸. In this sample, the normal to the main surface of the mineral particles is mainly parallel to the rotational axis of the sample. This allows particles to scatter the signal mainly along the vertical direction of the detector, which we analyze by integrating the signal within an angular sector of Δχ = 6°.

In a scanning SAXS experiment the whole width of the sample was covered by the measurement grid, with the grid step defined by the step size between measurements. In both horizontal and vertical directions, the size of the grid step was equal to the beam size. The maximum number of horizontal scanned lines in the grid was 3/25. The same SAXS scan was repeated after rotating the sample around its long axis [Fig. 2 ▸(*a*)]. The measured set of angles θ ranged between 0 and 180° with an angular step of 3°/4°. Therefore, the number of measured SAXS patterns for each sample of bovine bone was 2745/45 000.

In addition to the scattering data, X-ray attenuation data were acquired using a diode with an exposure time of 0.3 s/0.5 s. The total time for collecting a data set at BESSY was about 45 h, while for the presented data set measured at DLS the total time amounted to around 8 h.

**Figure 3:**
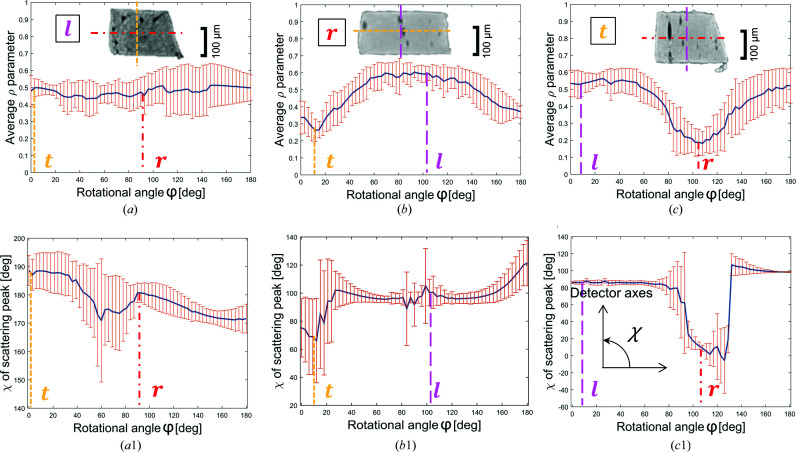
Macro- to nanostructure correlation. The plots show averaged values of the ρ parameter for the samples with the main axis aligned to the (*a*) longitudinal, (*b*) radial and (*c*) tangential directions of the samples. The values of the acquired ρ parameter at each rotational angle were averaged and the error bars indicate standard deviations. Dashed lines in each plot indicate the other two directions. (*a*1), (*b*1), (*c*1) Plots of the position of the maximum of the scattering intensity (χ), where χ denotes the azimuthal angle, as a function of the rotational angle φ.

In the scattering experiments the rotational axis 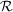 of the sample coincides with the long axis of our stick-like sample. Samples were prepared in such a way that the longest extension is aligned with one of the main directions of the femoral bovine bone, *i.e.* the longitudinal, radial or tangential direction [Fig. 1 ▸(*a*)]. To link the rotational angle θ of the measurement with the position of the sample in the Cartesian coordinate system defined by the longitudinal, radial and tangential directions, sinograms of the attenuation coefficients were analyzed on the basis of the known shape of sample cross sections. As an example, for the radial sample of Fig. 3(*b*) (see Section 4.1) the tangential direction was determined to correspond to ∼12°.

## 3. SAXS-invariant tomography

### 3.1. SAXS invariants and platelet thickness

The SAXS intensity in 3D reciprocal space can be written as 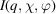 , where *q* is the length of the scattering vector [*q* = (4π/λ)sinθ, θ is half the scattering angle and λ is the wavelength of the incident radiation], χ the azimuthal angle measured with respect to an axis 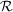 and φ the rotation angle around this axis 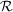 . When the specimen is rotated around the axis 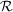 defined by 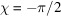 or 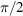 , the intensity in this direction stays unchanged since the rotation axis remains fixed during the rotation. In the case of bone where there is a strong electron density contrast between the mineral and organic phases, a two-phase model can be used to describe the small-angle scattering and 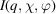 is taken to be proportional to the function 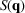 defined below. The proportionality constant between the two functions will depend on instrumental parameters, as well as on the squared electron density difference between the organic and mineral phases. The vector 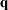 is defined by its length *q* and the two angles χ and φ.

We consider a two-phase model with a function 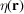 that is equal to 1 if there is a particle at position 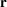 and is zero otherwise. Then the SAXS intensity will be proportional to 

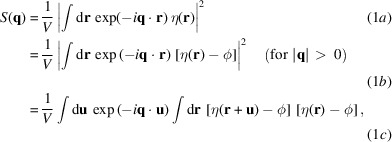

where *V* is the probed volume and ϕ denotes the spatial average of η that just equals the volume fraction of particles in the volume *V*. In equation (1*b*), note that the subtraction of ϕ has no effect on 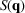 outside the origin of reciprocal space, 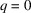 . However, the average of 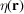 will formally generate a Dirac δ function at the origin that is practically invisible in SAXS. Therefore, the subtraction of the constant term ϕ ensures that the average of 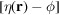 over the whole volume is zero and that there is no contribution of the Dirac δ function at 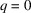 (which would otherwise contribute to the analytically calculated integral intensity). This is the usual procedure in the treatment of SAXS signals from two-phase systems, for example in solution scattering, and the spherical average of this expression is (Guinier & Fournet, 1955 ▸)

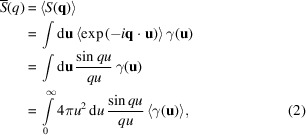

where the angle brackets denote the spherical average with respect to 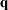 on the first two lines of the expression and with respect to 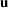 on the last line. The first expression comes because only the exponential term depends on 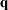 , and the second expression results from the averaging of the exponential term. In the last step, we rewrite the integration in spherical coordinates for the vector 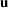 , and – since 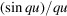 does not depend on angle – we remain with a single integral with respect to *u*, provided we replace γ by its spherical average with γ denoting the correlation function, 

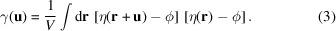




An inverse Fourier transform yields the expression for the spherically averaged correlation function (Guinier & Fournet, 1955 ▸):

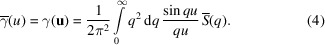

The consequence is the first SAXS invariant, the integral intensity

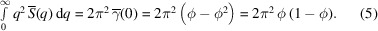

A Taylor expansion of the correlation function to the first order in *u* gives the second invariant, Porod’s law (Guinier & Fournet, 1955 ▸), where *S* is the total amount of particle interface in the volume *V*: 

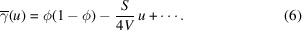

The Fourier transform then gives the limit of the function 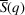 for large *q* as 

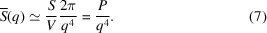

This has been used extensively [for a recent review, see Pabisch *et al.* (2013 ▸)] to determine an average thickness of particles through the parameter *T* defined as

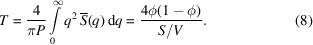

Here, ϕ and 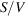 are the volume fraction and the surface per unit volume of the particles, respectively. For thin particles with thickness *W*, this is well known to correspond to (Pabisch *et al.*, 2013 ▸) 

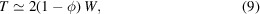

so that *T* roughly represents the mean particle thickness for a material with a particle volume fraction close to 50% (as in bone, for example).

The goal is now to generalize these expressions for integration along a rotation axis defined by the vector 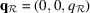 . We suppose that the specimen contains thin plates only. We first consider the contribution to the scattering by a single platelet oriented perpendicular to 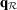 with a thickness *W*, and with breadth and length of *B* and *L*, respectively. Using equation (1*a*), the contribution of this particle to the total SAXS intensity can be written in Cartesian coordinates where *z* is along 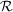 , yielding a well known result (Guinier & Fournet, 1955 ▸): 

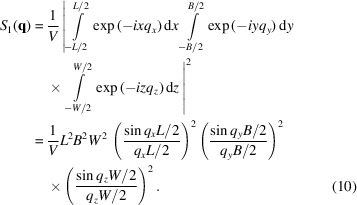

Using the fact that the function 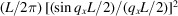 converges to the Dirac δ function when *L* gets very large, we obtain 

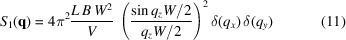

for *L* and *B* sufficiently large (in practical terms, larger than what SAXS would resolve in the relevant *q* range). Now, we define 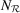 as the number of particles with their normal directions oriented within a small solid angle around 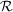 and we denote by 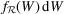 the corresponding thickness distribution [normalized so that 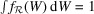 ]. Then the total intensity pointing in the 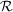 direction will be 

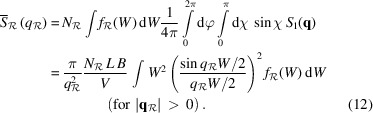

The angles are defined such that 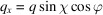 , 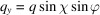 and 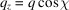 , and we have neglected possible interference effects between the particles. The volume fraction of all particles perpendicular to 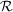 is 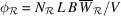 , where 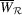 is the average thickness of the particles. Note that the same family of particles will also generate a similar scattering in the direction related to 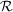 by an inversion symmetry with respect to the origin (meaning in the 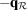 direction) but will not contribute to the scattering in any other direction.

Equation (12) can be expanded at large 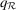 to give an analog to Porod’s law, 

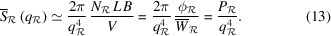

Moreover, the integrated intensity along the 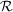 direction (starting at the origin of *q* space) will be proportional to the total volume of particles perpendicular to this direction, that is, proportional to 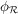 . The proportionality constant must be such that we recover equation (5) when summing over all possible directions. Here, we need to take into account that each plate-like particle scatters in two directions related by inversion symmetry, so that we are counting each family of particles twice when we integrate over all directions (hence the factor 1/2 in the equation below) to get 

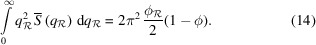

Indeed, when we sum this expression over all possible directions, and considering that 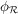 and 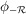 refer to the same family of particles, 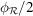 sums to ϕ, recovering equation (5). Taking the ratio of the integral intensity above and the Porod constant in the 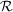 direction, 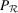 , we obtain in analogy to equation (9)


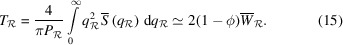




### 3.2. SAXS data evaluation

The acquired scattering intensity on the 2D detector, 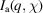 , was corrected considering X-ray attenuation and background subtraction according to 

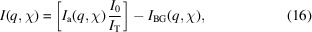

where 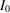 is the intensity of the incoming beam, 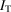 the transmitted intensity and 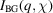 the background intensity. The angle χ is measured with respect to an axis 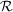 . 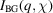 was obtained by averaging the scattering pattern of three measurement points of a scan which were outside the sample. Due to the fluctuation in the beam flux during the experiments at BESSY II, a further correction was applied to the measurements using background images for normalization.

In general, this intensity is not a voxel property, since scattering crucially depends on how the nanostructural elements, like the mineral particles in bone, are oriented with respect to the incoming beam. However, the scattering pattern does not change under sample rotation around the rotational axis, *i.e.* 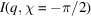 and 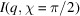 are independent of the specimen rotation around 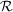 . Indeed, if the sample is rotated by an angle θ around 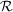 , the reciprocal space is also rotated by the same angle around the same axis. An alternative explanation which considers the nanostructure of bone is that by focusing only on the scattered signal around the rotational axis 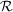 it is always the same subpopulation of mineral particles which contributes to the scattering signal. Taking into consideration the plate-like shape of the particles, this subpopulation consists of platelets with their normal oriented along the rotational axis [dark-gray particles in Fig. 2 ▸(*b*)].

For the calculation for the axial integrated intensity 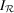 and the axial Porod constant 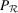 , an azimuthal integration of 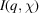 in a very narrow sector around 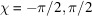 with an opening angle of Δχ was performed. Our choice of Δχ = 6° proved to be a good compromise to be, on the one hand, large enough to provide a robust value for 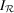 and, on the other hand, small enough to ensure that 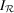 is independent of the rotational angle 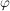 . The *q* ranges in the evaluation were 0.2–3 nm^−1^/0.1–2.6 nm^−1^.

Besides the reconstruction of the 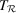 parameter, a second parameter (ρ parameter), which describes the mutual alignment of the mineral particles (Pabisch *et al.*, 2013 ▸), was evaluated. To obtain the ρ parameter the SAXS patterns are radially integrated, yielding the scattered intensity as only a function of the azimuthal angle, 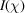 . The function 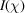 displays two peaks separated by 180° on top of a constant background. The ρ parameter is defined as 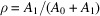 , where 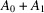 denotes the area under the curve 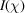 including the constant background, and 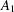 the area under the peaks only. Consequently, the ρ parameter takes values between 0 and 1, where 0 corresponds to a random mutual alignment and 1 to a perfect alignment of all the mineral particles.

All the SAXS data analysis was performed using the software packages *DAWN* (Basham *et al.*, 2015 ▸; Filik *et al.*, 2017 ▸) and *DPDAK* (Benecke *et al.*, 2014 ▸). In order to visualize the ρ parameter as a function of the rotational angle (Fig. 3), the values of the ρ parameter acquired at a specific rotational angle were averaged.

### 3.3. Tomographic reconstructions

**Figure 4:**
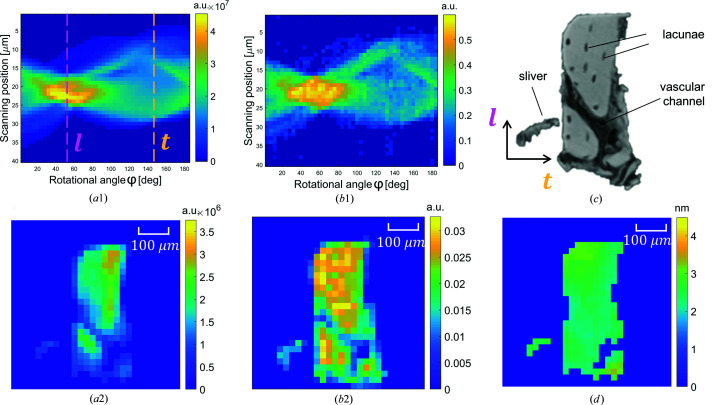
Comparisons between reconstructions, using different techniques, of the region at the interface between two fibrolamellar units. (*a*1) A sinogram of the integrated intensity achieved from SAXS measurements. Here the longitudinal and tangential directions are marked by dashed lines. (*b*1) A sinogram of the quantity 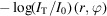 . (*c*), (*a*2), (*b*2) Reconstructions from, respectively, the µCT, SAXS and synchrotron CT measurements. (*d*) Reconstruction of the 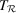 parameter map.

The essence of tomography is to reconstruct the bulk properties of a sample when only projection data are available. Typically, the different projections are obtained by scanning and rotating the sample. In the parallel-beam geometry applied in SAXS tomography experiments, reconstruction is a 2D problem defined by the slice of the sample which is scanned perpendicular to the rotational axis 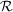 . In mathematical terms, projection data *p* are obtained at different scanning positions *r* and different rotational angles φ and are usually plotted as a function of these two variables, 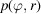 , as a sinogram [see *e.g.* Figs. 4(*a*1) and 4(*b*1) in Section 4.2]. From these input data a material property 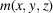 can be inferred, where *x* and *y* are coordinates in the coordinate system of the sample and *z* is assumed fixed, since we consider here only the reconstruction of a 2D slice of the sample.

The reconstruction problem is solved when two requirements are fulfilled:

(i) The material property is a scalar property of the ‘voxel’ representing a small material volume and, in particular, does not depend on the angle φ under which the voxel is measured. The X-ray attenuation coefficient, 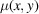 , is such a voxel property.

(ii) The material property is an additive quantity, *i.e.* 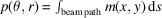 . 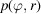 is called the Radon transform of 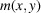 . In the case of X-ray attenuation, the Beer–Lambert law ensures that the logarithm of the measured transmitted intensity is the Radon transform of the attenuation coefficient of the material.

For each synchrotron experiment, three reconstructions were performed. From the SAXS experiments, (i) the axial integrated intensity 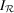 and (ii) the axial Porod constant 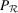 were reconstructed. From the X-ray attenuation experiments (synchrotron CT), (iii) the attenuation coefficient 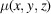 was reconstructed starting from measurements of the transmitted intensity, specifically 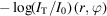 . For the reconstruction, a filtered back-projection (FBP) algorithm was used (Thorsten, 2011 ▸) as implemented by the MATLAB function *Iradon*. Due to the virtually parallel beam geometry, the reconstruction could be performed in ‘slices’ of the sample, where the slice has a normal identical with the rotational axis 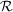 and a thickness defined by the beam size.

To perform an FBP, we need to specify the type of interpolation and high-pass filtering that accomplish the mapping from the polar coordinate system of the sinogram to the Cartesian one after reconstruction. From the options offered by the *Iradon* function, a spline interpolation and a Ram–Lak filter yielded the most satisfying reconstruction results. The reconstruction quality was tested by projecting the result of the reconstruction (*i.e.* by performing a Radon transform) and calculating the mean-squared error between these projected data and the original measurement data. To avoid spurious results for the *T* parameter 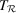 as a result of a division of two values close to zero, a threshold value for the axial integrated intensity 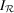 was introduced as 16% of the maximum value of 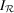 after reconstruction. All 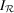 values below this threshold were set to zero. Our choice of the threshold rendered the outer shape of the sample close to results from µCT measurements (Section 2.2). A sensitivity analysis showed that the exact value of the threshold has a negligible influence on the reported results.

The attenuation coefficients 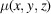 were reconstructed after normalization of the data to correct for variations in the beam intensity. The data were first thresholded by setting all values of the sinogram data 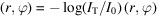 which are smaller than 6% of the maximum value equal to zero. For the normalization a factor was used which was obtained by averaging 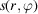 for a fixed rotational angle. The time for the reconstruction of each slice (*i.e.* fixed *z* coordinate) for integrated intensity, Porod constant and attenuation coefficient was below one second with a standard PC using FBP.

### 3.4. Spatial correlations

To allow a spatial correlation between the bone microstructure (vascular channels, osteocyte lacunae) and the mineral nanostructure, distance transforms were used. After binarizing the µCT image and defining the voxels in the digital image that belonged to vascular channels/osteocyte lacunae, the distance transform assigns each bone voxel in the image the shortest distance value to the defined objects. Calculations were performed in MATLAB using the function *bwdistsc*. Image registration (using the MATLAB function *imregtform*) between the µCT image and the 3D reconstruction of the attenuation coefficients from the synchrotron experiment was employed to map microstructure information (distance transforms) on nanostructure information (map of the *T* parameter).

## 4. Results

### 4.1. Correlation between mineral nanostructure and sample macrostructure

In a first step, we analyzed anisotropies of the mineral nanostructure in relation to the macroscopic coordinate system of the bovine femur defined by the longitudinal, radial and tangential directions [Fig. 1 ▸(*a*)]. The nanostructural anisotropy was assessed by the ρ parameter (see Section 3.2). The analyzed sample slices (*i.e.* cross sections through the sample with a thickness of the beam size) had their normals (which are identical to the rotational axis 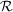 ) in the direction of the longitudinal, radial and tangential directions [respectively, Figs. 3 ▸(*a*), 3 ▸(*b*) and 3 ▸(*c*)]. For each rotational angle, a ρ parameter was calculated as the average over all measurement points in the slice [Figs. 3 ▸(*a*), 3 ▸(*b*) and 3 ▸(*c*)]. Different dependencies as a function of the rotational angle are observed: an almost constant curve for the longitudinal sample, while the radial and tangential samples each exhibit a minimum. These minima correlate well in position with the macroscopic directions, demonstrating that the preferred orientations of the mineral nanostructure as quantified by the ρ parameter align with the macroscopic coordinate system. The majority of mineral particles are aligned with their long axis along the longitudinal direction. Fig. 3 ▸(*a*1) (longitudinal sample) shows that the scattering intensity 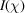 is a maximum at an azimuthal angle χ of about 180°, independent of the rotational angle φ, *i.e.* perpendicular to the rotation axis, and therefore 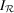 is low in the longitudinal sample. Radial samples are considered to exhibit the most effective scattering power for our SAXS tomography approach, with a position of the maximally scattered intensity near χ = 90° at most of the rotational angles φ and, therefore, a large value for 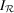 [Fig. 3 ▸(*b*1)]. The majority of particles in the radial sample are oriented such that their long axes are perpendicular to the long axis of the sample and these particles contribute to the scattering signal mainly along the direction of partial integration (vertical axis of the detector in our setup), as described in Section 2.3.

### 4.2. SAXS tomography and 3D *T* parameter map

The experimental setup allows one to obtain 3D maps by performing an independent reconstruction of sample slices (*i.e.* slices of a height equivalent to the beam size and oriented normal to the rotational axis 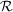 ) and piling these slices up after reconstruction to obtain the full 3D information. Fig. 4 ▸ shows representative data for a sample slice that includes a vascular channel. In the µCT image of Fig. 4 ▸(*c*), not only can the vascular channel be clearly discerned, but osteocyte lacunae are also visible as small dark ellipsoids. A bone sliver close to the lower left corner of the sample, accidentally produced during cutting, additionally helped in the image registration of the different measurements.

Figs. 4 ▸(*b*1) and 4 ▸(*a*1) show 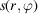 from the absorption measurement and the projections of the axial integrated intensity from the scattering experiment as sinogram plots (*i.e.* as a function of the rotational angle φ and the scanning position *r*). The shape of the sample contributes substantially to the values in the sinogram, with the values being highest when the sample is viewed along its diagonal. The corresponding reconstructions for the attenuation coefficient 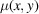 and the axial integrated intensity 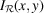 are shown in Figs. 4 ▸(*b*2) and 4 ▸(*a*2), respectively. A comparison between the µCT image and the reconstructed attenuation coefficient shows that the synchrotron experiment allows a reliable reconstruction of the sample shape (including the sliver) and larger internal structures like vascular channels. However, the lacunae are too small to be visible.

Having reconstructed data not only for the axial integrated intensity 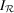 but also for the axial Porod constant allows the determination of the thickness 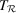 of mineral particles which have their normal parallel to the rotational axis 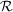 . The resulting map [Fig. 4 ▸(*d*)] shows spatial gradients in the particle thickness, with values above 3 nm in the upper left corner and values below 2 nm close to the vascular channel.

**Figure 5:**
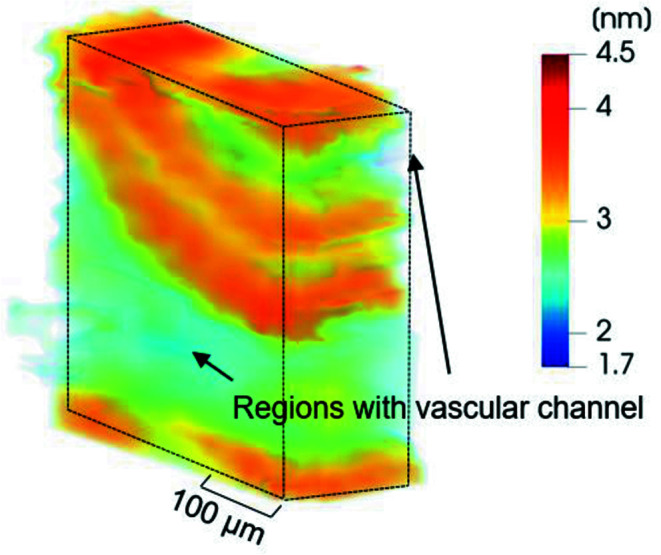
Structural characterization of the radial sample, showing a 3D reconstructed map of the 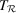 parameter for the sample with its main axis aligned to the radial direction of the bovine femur.

Repeating the evaluation for all measured sample slices and arranging the slices in the correct spatial order results in the 3D 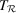 parameter map shown in Fig. 5 ▸. The values of the 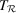 parameter vary between 1.7 and 4.5 nm, where regions close to the vascular channels display low values of 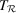 (region in light blue). The highest values of 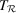 were found within two band-like structures (regions in orange/reddish hue), with the thicknesses of the bands roughly 40 µm and a separation between them of about 15 µm.

### 4.3. Spatial correlation between micro- and nanostructure

**Figure 6:**
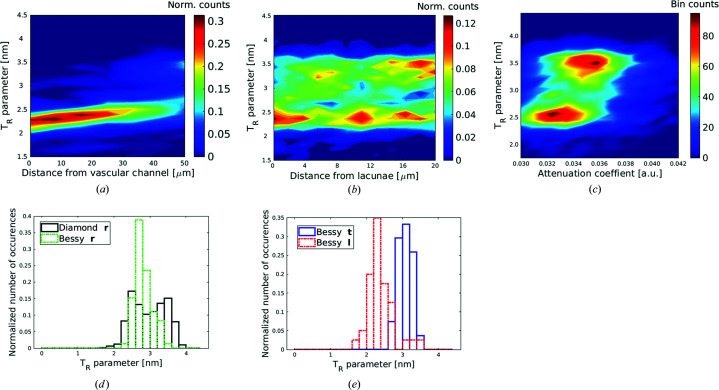
Analyses of the reconstructed 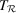 parameter values. (*a*) The 3D distance transformation of the 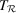 parameter map taking as reference the voxels containing the blood vessels. (*b*) The 3D distance transformation of the 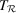 parameter map taking the lacunae as reference points. (*c*) The correlation between the 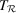 parameter and attenuation coefficients reconstructed from synchrotron CT measurements. (*d*) A normalized frequency plot of the 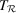 parameter distribution in the two radial samples. (*e*) A normalized frequency plot of the 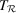 parameter distribution in the tangential and longitudinal samples.

The experimental design, with the same X-ray beam used to conduct both an absorption and a scattering experiment, allows a straightforward evaluation of spatial correlations between different structural quantities. In Fig. 6 ▸(*c*) the attenuation coefficient and the 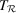 parameter for identical voxels in the 3D reconstructions are plotted. While 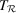 reflects the nano­structural thickness of the mineral particles, the attenuation coefficient provides information about the local mineral content. The data points are concentrated in two regions of the plot: thin particles ( 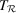 ≃ 2.5 nm) and low mineral content, and thicker particles ( 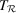 ≃ 3.5 nm) and higher mineral content.

Next, the mineral nanostructure is correlated with microscopic features of the fibrolamellar bone. In Fig. 6 ▸(*a*) the 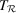 parameter is plotted as a function of distance from the vascular channel. Bone close to the vascular channel exhibits particularly low values of the 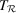 parameter. No clear spatial correlation can be detected when looking at the particle thickness as a function of the distance from the osteocyte lacunae [Fig. 6 ▸(*b*)].

In the frequency plots of Fig. 6 ▸(*d*) the normalized number of occurrences of the 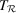 parameter in both analyzed radial samples is represented. While data from the two samples with a smaller imaged volume show only one peak at relatively low values of the 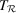 parameter with a mean value of 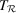 = 2.81 nm and a standard deviation (SD) of 0.23, the analogous plot of the other radial sample with the larger imaged volume exhibits two peaks of the 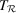 parameter, at 2.60 and 3.40 nm, as calculated with a Gaussian mixture model. Fig. 6 ▸(*e*) shows frequency plots for the longitudinal and tangential samples. For the longitudinal sample the obtained 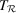 parameter is relatively low (mean value 2.39 nm and SD 0.36) compared with the tangential sample (mean value 3.09 nm and SD 0.19).

## 5. Discussion and conclusions

In this proof-of-concept study on a mineralized tissue, we have demonstrated that 3D nanostructural information about mineral particle characteristics can be obtained using a new form of SAXS tomography. Our strategy was to define a quantity with contributions adding up from all the voxels that the X-ray beam passes through during the experiment, which is a voxel property independent of its orientation in relation to the X-ray beam. This strategy then allows the use of standard reconstruction methods (Thorsten, 2011 ▸) to transform the projected data into a 3D map. We have demonstrated the feasibility of this procedure for the *T* parameter, which is calculated as the ratio of two scattering invariants, the integrated intensity and the Porod constant, and which is an important indicator of tissue maturity and its mechanical performance.

A prerequisite of our approach is that the evaluated scattering signal remains unchanged during the rotation of the sample. This rotational invariance is only fulfilled for scattering around the rotational axis, and consequently only slightly more than 3% of the detector information is used in the evaluation. This restriction of the evaluation in reciprocal space corresponds to selecting a subpopulation of the mineral particles in real space. Only mineral particles with a normal parallel to the rotational axis are considered in the evaluation and are, therefore, described by the reported 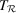 parameter (which is, for this reason, denoted with a subscript). This specificity of our method for the particle orientation carries implications for its usefulness. In tissues with a preferred matrix orientation, the investigation can focus on the mineral particles which are embedded in the matrix in conformance with its preferred orientation. The type of bone used in this study – fibrolamellar bone – falls into this category, with the fibrous collagenous matrix preferentially orientated along the axis of the long bone. Similarly to methods like polarized light microscopy, Raman spectroscopy and second-harmonic generation microscopy, which exploit orientational interaction effects with collagen, our method is able to detect variations in the alignment of mineral particles by yielding a detectable scattering signal only if particles are ‘in plane’, *i.e.* if the normals to their plate-like surfaces are parallel to the rotational axis.

SAXS is an important technique to characterize the nanostructure of inorganic–organic hybrid materials (Peterlik & Fratzl, 2006 ▸), and SAXS tomography may become a powerful way of mapping nanostructure variations in three dimensions within macroscopic specimens. Indeed, our approach is not limited to biological materials like bone. While our equations were derived for plate-like inclusions, they are valid more generally, and 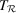 is then an average chord length of the inclusions measured in the direction 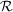 . Unfortunately, Porod’s chord-length measure does not directly describe a particle thickness, as in the case of platelet-shaped inclusions, but it still represents an interesting size characteristic of any nanostructured two-phase system.

An issue that has to be considered when studying biological materials with synchrotron radiation is the damage caused to the sample by the radiation. While radiation damage in X-ray tomographic approaches is known to affect the mechanical properties of bone strongly, mostly by the degradation of collagen (Barth *et al.*, 2010 ▸), the shape of mineral particles is less affected. Although we cannot exclude the influence of radiation damage, we did not observe a change in the SAXS intensity from the mineral over time scales corresponding to an experiment.

Our experimental setup allows the combination of several methods using the same X-ray beam as the probe. In the current study the beam was used to measure the absorption coefficient, which provides information about the local calcium content of the bone, together with the scattering signal. In a similar way, the combination of methods could be extended to include chemical analysis [*e.g.* using X-ray fluorescence (Lange *et al.*, 2011 ▸)]. With measurements based on the same incoming beam, the registration of different image data and the evaluation of the spatial correlation between different physical quantities [like the 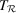 parameter and Ca content, see Fig. 6 ▸(*c*)] are straightforward.

An advantage of our approach is the strongly reduced effort required for both the experimental and computational work compared with tensor tomography, when a specific parameter like the mineral particle thickness is sought. In the experiment, a single rotation of the sample is sufficient to obtain reconstructable information about the nanostructural thickness. As a result, the size of the data set for reconstruction is reduced and is, therefore, less demanding on the RAM of the reconstruction computer. In addition, the reduction in the dimensionality of the reconstruction problem allows the use of standard reconstruction algorithms, which have been optimized over the past few decades. Computational time can be saved by performing the preparatory evaluation of SAXS parameters during the measurement itself.

We tested our method on samples of fibrolamellar bovine bone. The preparation of a stick-like sample oriented in the radial direction and an imaged volume of 250 µm in this radial direction allowed us to image all the different layers within a complete fibrolamellar unit. As described by Magal *et al.* (2014 ▸), starting from a vascular channel the succession of layers are first lamellar bone around the channel, then a parallel-fibered layer, the primary hypercalcified layer in the center of the fibrolamellar unit, and then again the succession of a parallel-fibered layer and lamellar bone, before reaching the next layer with vascular channels. Based on the µCT image of our sample, we observed vascular channels close to the top of the sample and in the lower part approximately 180 µm apart. Due to its branching, the lower vascular channel occupies a substantial area within a cross section of the sample [Fig. 4 ▸(*c*)].

Using the vascular channels as spatial references leads to the following interpretation of the 3D 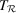 parameter map of Fig. 5 ▸: the lamellar bone around the vascular channels displays low values of the *T* parameter, also clearly observable in the spatial correlation plot [Fig. 6 ▸(*a*)]. The highest values of the 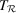 parameters are found in two curved layers with a thickness of 30 to 40 µm, with the lower layer thicker than the upper one. These two layers are interleaved with a thinner layer (∼15 µm thick), which comprises thinner mineral particles. The location of the layers and their thicknesses correspond exactly to the core of the fibrolamellar unit, with the central hypercalcified layer with a lower 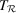 parameter separating the layers of parallel-fibered bone with thick particles. The correlation plot of Fig. 6 ▸(*c*) shows not only that the lamellar bone has thinner mineral particles but also that the mineral content is lower compared with the parallel-fibered bone.

The 3D map of the *T* parameter provides new insights into the arrangement of the fibrolamellar units. While the lamellar bone around the vascular channel at the bottom results in a clear separation between the fibrolamellar units, the lamellar bone on top is quite localized around the more isolated vascular channel, which results in a more continuous transition between the two fibrolamellar units at the top. In plexiform (fibrolamellar) ovine bone, a similar correspondence of the *T* parameter and structural organization was found, *i.e.* relatively high *T* parameters in regions with a higher degree of structural organization and lower *T* parameters in the more woven-like areas (Kerschnitzki *et al.*, 2013 ▸). Testing different subpopulations of mineral particles showed differences in the 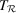 parameter [Figs. 6 ▸(*d*) and 6 ▸(*e*)]. While these measurements were performed on differently oriented samples, it is straightforward to test different subpopulations in the same sample by rotating the sample around more than one rotational axis 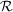 or by reconstructing the full 3D scattering pattern (Liebi *et al.*, 2015 ▸; Schaff *et al.*, 2015 ▸). In addition, our study is limited by a low sample number. A systematic investigation of several fibrolamellar units in different locations of bovine bone and in different individuals would allow a quantitative assessment of biological variability.

The experimental approach proposed in this study provides much more information about the sample than is actually used for the reconstruction. One might also implement the idea of virtual rotation axes, originally proposed by Schaff *et al.* (2015 ▸), into this simplified treatment of SAXS data where only invariants are reconstructed. This would probably make the reconstruction of particle sizes with many orientations more effective. Furthermore, the recent past has proved that important progress in imaging techniques occurs on the computational side of the process. For our problem we see a substantial potential to exploit information from the beam profile and the availability of two data sets (scattering and attenuation) in order to improve the reconstruction result in combination with algebraic reconstruction tools (Hansen & Jørgensen, 2018 ▸).

Finally, the success of a new method is always linked to interesting applications. In investigating mineralized tissues, the most pressing problems encountered are posed by bone diseases. While mineralization disorders are often characterized in terms of the amount of mineral incorporated in the bone (Roschger *et al.*, 2008 ▸), our method would allow a mapping of structural disorders of the mineral nanostructure.
